# Monitor for COVID-19 vaccine resistance evolution during clinical trials

**DOI:** 10.1371/journal.pbio.3001000

**Published:** 2020-11-09

**Authors:** David A. Kennedy, Andrew F. Read

**Affiliations:** 1 Center for Infectious Disease Dynamics, Department of Biology, The Pennsylvania State University, Pennsylvania, United States of America; 2 Department of Entomology, The Pennsylvania State University, Pennsylvania, United States of America

## Abstract

Although less common than the evolution of antimicrobial drug resistance, vaccine resistance can and has evolved. How likely is it that COVID-19 vaccines currently in development will be undermined by viral evolution? We argue that this can be determined by repurposing samples that are already being collected as part of clinical trials. Such information would be useful for prioritizing investment among candidate vaccines and maximizing the potential long-term impact of COVID-19 vaccines.

## Abstract 2

Will a safe and efficacious COVID-19 vaccine be undermined by vaccine-resistance evolution? Rather than take a wait-and-see approach, this Perspective article argues that this question can be answered even before a vaccine is licensed, by repurposing samples already being collected in clinical trials.

A safe and effective vaccine against COVID-19 would go a long way towards helping society return to its pre-pandemic normal. According to the World Health Organization, at least 198 COVID-19 vaccines are currently in the development pipeline, with 44 currently undergoing clinical evaluation [1]. That evaluation is, rightly so, focused on safety and efficacy. Here, we advocate for moderate additional effort during clinical trials to collect and publish data that can inform the risk of resistance evolution.

Much like antimicrobial drug resistance, vaccine resistance can and does evolve [2]. When it does evolve, vaccine resistance is achieved through mechanisms such as serotype replacement [3], antigenic change [4], or increases in disease severity [5]. However, for many vaccines, the evolution of resistance has never occurred [6]. For example, the measles vaccine has been widely used for decades without the virus ever evolving the ability to transmit through vaccinated hosts. Similarly, smallpox was completely eradicated, in large part due to vaccination that viral evolution failed to overcome. In contrast, *Streptococcus pneumoniae* quickly evolved resistance to the pneumococcal conjugate vaccine (PCV7), necessitating the development and deployment of a new vaccine, PCV13 [7]. Recently, the features that are critical to delaying the evolution of vaccine resistance have been described [6]. Here, we argue that by repurposing standard samples from COVID-19 clinical trials, the potential for vaccine resistance can be assessed even before vaccine licensure.

To our knowledge, all documented cases of vaccine resistance can be attributed to the absence of at least one of three key features that most vaccines possess: 1) the vaccine induces an immune response that protects hosts by targeting multiple virus epitopes simultaneously, thereby generating redundant and evolutionarily-robust protection, 2) the vaccine suppresses pathogen growth within hosts and stops transmission from vaccine-protected hosts, and 3) the vaccine-induced immune response protects against all circulating serotypes of the target pathogen. When feature 1 is present, resistance would likely require the appearance of multiple mutations, as opposed to just one, on the same genetic background. When feature 2 is present, little pathogen diversity would be generated during pathogen growth within vaccinated hosts, and the effects of selection on any resistance mutations that arose would be minimal. When feature 3 is present, new virus variants would need to be generated before resistance could be a problem, since vaccine resistance does not pre-exist. Combined together, these three features make the probability of resistance emergence vanishingly small [6].

It is important that the probability of resistance evolution be small because vaccine resistance can negatively impact public health. While antimicrobial drugs can be tailored to individual patients at the time of treatment, the choice of which vaccine to administer must be made well in advance of pathogen exposure. Should vaccine resistance emerge in the weeks, months, or years between vaccination and exposure, a vaccinated individual could be left unprotected. Should resistance become widespread and common, entire vaccination campaigns could retroactively be rendered ineffective. Moreover, since pre-existing antibodies frequently interfere with vaccine efficacy [8], we cannot assume that a new vaccine would be capable of restoring protection. Additionally, a large fraction of COVID-19 candidate vaccines target the spike protein of the virus or the receptor binding domain of the spike protein [9], and so the evolution of vaccine resistance against one vaccine could simultaneously undermine others, an outcome referred to as ‘collateral’ or ‘cross’ resistance in the case of antimicrobial drugs.

To avoid being caught off guard by the evolution of vaccine resistance, standard samples from clinical trials can be repurposed to assess the risk of resistance evolution even before a vaccine is licensed (Fig 1). First, blood samples are collected during almost all COVID-19 clinical trials to quantify individual responses to vaccination through antibody titer and serum neutralization tests. We propose that in addition to performing these tests, blood samples also be used to quantify the redundancy of immune protection generated by candidate vaccines [10,11]. Since redundant immune protection delays the evolution of vaccine resistance, much the same as combination drug therapy delays the evolution of antibiotic resistance, it is critical that vaccination induces immune responses against multiple non-overlapping viral epitopes. For SARS-CoV-2, as in other systems, resistance has already been shown to evolve quickly against monoclonal neutralizing antibodies relative to combinations of these antibodies [12]. Although yet to be shown for SARS-CoV-2, diverse T-cell responses can similarly delay resistance evolution [7]. Therefore, quantifying the redundancy of immune protection generated by vaccination is key information for determining the likelihood of resistance evolution.

**Fig 1:**
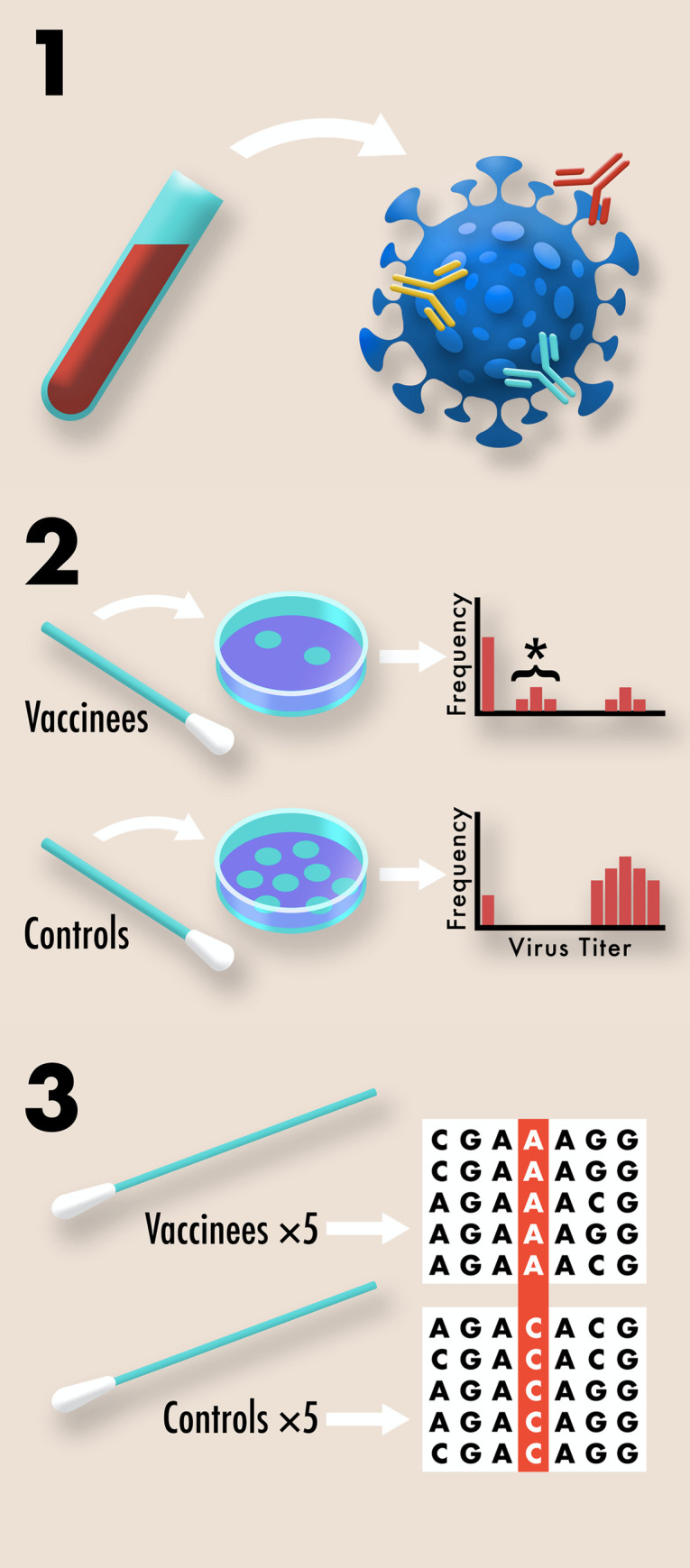
Schematic illustrating three ways that standard samples from COVID-19 clinical trials can be repurposed to assess the risk that vaccine resistance will evolve. 1. The complexity of B-cell and T-cell responses can be measured using blood samples [10,11]. Different neutralizing antibodies are depicted above in different colors. More complex responses indicate more evolutionarily robust immunity. 2. The effect of vaccination on transmission potential can be assessed by collecting viral titer data using routine nasal swabs. Plaque assays from multiple vaccinated and control individuals are compiled into a histogram. Undetectable viral titers suggest little or no transmission potential, due to either complete immune protection or the absence of exposure. High viral titers suggest high transmission potential due to the absence of a protective immune response. Intermediate viral titers, marked above with an asterisk, suggest moderate transmission potential due to partial vaccine protection. Intermediate titers indicate an increased risk for resistance evolution since pathogen diversity can be generated within hosts and selection can act during transmission between hosts. 3. Pre-existing variation for vaccine resistance can be assessed by recovering genome sequences from nasopharyngeal swabs of symptomatic COVID-19 cases included in the study. In a placebo controlled, double blind study, any significant differences in the genome sequences of samples from vaccinated and control individuals would suggest at least partial vaccine resistance.

Second, many COVID-19 vaccine clinical trials collect weekly nasal swabs or fecal samples from vaccinated and control individuals to quantify vaccine protection against infection. We propose that these samples also be used to collect viral titer data as indicators of transmission potential. Strongly suppressing pathogen transmission through vaccinated hosts is key to preventing the spread of partial resistance should it arise, since it reduces the opportunities for selection to act [6]. While viral titer data are imperfect measures of transmission, they are a readily collectible proxy. Note that extra effort to collect higher quality transmission data may also be justifiable given the value of transmission data for optimizing vaccine distribution [13].

Third, many COVID-19 clinical trials collect nasopharyngeal swab samples from symptomatic vaccinated and control individuals to confirm SARS-CoV-2 as the causative agent of illness. We propose that viral genome sequences be generated from these swabs to look for evidence of vaccine-driven selection. For example, differences in allele frequencies between the viral genomes collected from vaccinated and control individuals would indicate selection [14], while simultaneously alleging a genetic basis for resistance [2, 3]. If such evidence were seen during a clinical study, as it can be [3], it would strongly indicate the potential for resistance to evolve.

A safe and effective COVID-19 vaccine is a priority, and it is urgently needed. Given this, we are not advocating to delay the release of a COVID-19 vaccine that is safe and efficacious even if there is a high likelihood that resistance will evolve against it. Rather, we are advocating that all vaccines be assessed as early as possible for the likelihood they will drive resistance evolution. As we explain above, this assessment can be conducted in a controlled manner during clinical trials, rather than first waiting for promising trial results to melt away after a vaccine is licensed.

For other diseases, vaccine failure due to pathogen evolution has occurred both during clinical trials [14] and after licensure [2]. We therefore suggest that the risk of resistance be used to prioritize investment among otherwise similarly promising vaccine candidates. If all first-generation vaccines are at appreciable risk of being undermined by virus evolution, it will be important to continue additional COVID-19 vaccine development following the discovery of a first, safe and efficacious vaccine. Predicting when and how resistance will be likely to evolve will give important insight into what needs to be monitored in phase IV studies after vaccine roll-out [3,7].

The world needs a COVID-19 vaccine urgently, just as the world previously needed drugs against tuberculosis and human immunodeficiency virus (HIV). It is tempting to leave evolutionary concerns until after a vaccine is introduced. But as we saw in the case of tuberculosis and HIV, the evolution of resistance can quickly undermine newly discovered interventions. By learning from solutions to previous evolutionary challenges, we can do better for COVID-19.
